# Supplementary material for: Designing tuberculosis elimination framework through participatory processes in Ethiopia: Results from stakeholders’ discussions
Source: PLoS One. 2025 Mar 10;20(3):e0318209. doi: 10.1371/journal.pone.0318209 (PMC11892823; doi:10.1371/journal.pone.0318209)
Supplement: S1 Table — indicates the coordinates of the study districts to prepare their maps, their population, health facilities and TB burden. (DOCX) [file pone.0318209.s001.docx]

**S1 Table. Profile of study districts.**

| Study districts | Catchment area Population | Number of Public hospitals | Number of Health Centers | Number of Private Hospitals | Number of Health Posts | Number of Health extension workers | Number of Kebeles | Number of House Holds | Number of HDA (if any) | TB CNR (2014) | Coordinates |
| --- | --- | --- | --- | --- | --- | --- | --- | --- | --- | --- | --- |
| Gedeb Hassassa --Oromia | 292, 569 | 0 | 8 | 0 | 25 | 66 | 25 | 60952 | 1106 | 130/100,000 | 7°10′N 39°10′E |
| Bolosso Bombe --SNNP | 118,913 | 01 | 03 | 0 | 21 | 65 | 21 | 24267 | 780 | 132/100,000 | 7°08′15.1"N 37° 34'54.1"E. |
| Jabi Tahinan--Amhara | 295578 | 1 | 12 | 0 | 49 | 85 | 49 | 73895 | 1281 | 140/100,000 | 10°50′N 37°10′E |
