# Supplementary material for: Designing tuberculosis elimination framework through participatory processes in Ethiopia: Results from stakeholders’ discussions
Source: PLoS One. 2025 Mar 10;20(3):e0318209. doi: 10.1371/journal.pone.0318209 (PMC11892823; doi:10.1371/journal.pone.0318209)
Supplement: S2 Table — indicates the template used by the researcher as a question guide and compiled the interview opinions and information. While brainstorming, detailed notes were captured in the transcription column. There is a discussion row where question guides were set. After the end of the discussion, the researchers noted down the code in the code column and summarized categories in the theme column. (DOCX) [file pone.0318209.s002.docx]

**S2 Table. Question guide template for the experience of MRC tailored feasibility and piloting.**

| **Transcription/field notes** | **Codes** | **Themes** | **Overall, Them** |
| --- | --- | --- | --- |
| **Discussion Topics** | | | |
| Q1. What do you feel about DeMONSTRATE TB study? | | | |
| Q2. How do you express your level of support for the study? | | | |
| Q3. What do you expect from the study? | | | |
| Q4. What other issues should be taken into consideration during this study? | | | |
| **West Arisi zonal CDC, Oromia** | - Outstanding initiative - Honored to support, - Revolutionizing TB - Screening, diagnosis, - patient care - TB elimination goal, - TB-free world, - Advancing efforts to eliminate TB, - Remarkable opportunity, - Important local evidence, - Benefit the community, - Unique challenges and opportunities, - Inform decision-making, - Drive positive change | ***Government interest toward local evidence*** | **Looking for local evidence compulsory for TB elimination** |
| The DeMONSTRATE-TB study is an outstanding initiative that we are honored to support. |  |  |  |
| Our ambition goes beyond simply contributing to a research project. This study is an intervention that holds the promise of revolutionizing the current practice of TB screening, diagnosis, and linking patients to treatment and care. |  |  |  |
| Our goal is to achieve the Elimination goal and bring us closer to a TB-free world. As the head of the West Arisi zonal CDC, I recognize the crucial role that this study plays in advancing our efforts towards eliminating TB. It is a remarkable opportunity to work towards a brighter future, and we are excited to be a part of it |  |  |  |
| **Bolosso Bombe District Head, Wolyta** |  |  |  |
| The study being conducted provides a valuable opportunity for our district to gather important local evidence that can ultimately benefit the community as a whole. By participating in the study, we can better understand the unique challenges and opportunities that exist in our local area and use this knowledge to inform decision-making and drive positive change. |  |  |  |
| **Wolyta Zone Health Buraeu CDC** |  | ***Homogeneity of officials' commitment across the regions*** |  |
| We are fully committed to ensuring the success of this study project. We believe that the package of interventions and resources offered by the project team is excellent, and we are thrilled to be able to support it. Our team is dedicated to going above and beyond to help achieve the goals of this project, and we are excited to enhance our commitment to this endeavor. By working together and leveraging our collective strengths, we are confident that we can help make this study project a resounding success. |  |  |  |
| **West Gojam, Amhara** |  |  |  |
| As the government, we recognize and accept our primary responsibility as the owner of the TB elimination plan. It is our duty to ensure the successful implementation of the program and we cannot delegate or transfer this responsibility to any third party. However, we greatly appreciate the support and contribution from various stakeholders in the fight against TB. This includes the valuable research conducted in this field, which plays a crucial role in enhancing our understanding and improving our strategies for TB prevention and treatment. Such support is highly valued and encourages us to further strengthen our efforts towards TB elimination-RHB representative. | - Enhanced commitment, - Collaboration and leveraging collective strengths. - Dedicated team, - Government's responsibility for TB elimination - Appreciation for support, - Encouragement to strengthen efforts, - Commitment to project success, - Belief in excellent interventions - Value of research |  |  |
| **West Arisi zonal CDC in Oromia** | - Importance of involvement of officials - involving the community at multiple levels - Sense of ownership - Procedural steps - Need of obtaining formal permission - Consequences of not abiding with system - Role of the health managers - Need of community engagement - Understanding of study objectives - Understanding of study outcomes - Active participation and contribution - Empowerment of the community - Need of active engagement - Need of community awareness - Unique perspectives, knowledge, and resources of community - Sense of ownership and collaboration - Enhancing intervention - Effectiveness and sustainability. | **The importance of community engagement** |  |
| Being involved from officials and the community at multiple levels is very important when it comes to conducting a study. This is because it not only helps to take ownership of the study but also ensures that proper procedural steps are followed before obtaining formal permission to proceed. Our experience has shown that studies which do not follow the necessary steps, such as obtaining approval from regional research approving bodies in addition to the national IRB, can be stopped. In fact, the head of the West Arisi zonal CDC in Oromia has been instrumental in ensuring that all studies conducted in our region follow these procedural steps. |  |  |  |
| **West Gojam, Amhara** |  |  |  |
| Community engagement is not an optional endeavor, but rather a mandatory action that should be implemented right from the outset of conducting a tuberculosis (TB) study within a community. It is crucial for the community to have a comprehensive understanding of the objectives and potential outcomes of the study. This awareness enables them to actively participate and contribute significantly in every phase of the intervention -Woreda representative |  |  |  |
| The active engagement and awareness of the community empower them to play a vital role and make substantial contributions throughout every phase of the intervention. By actively participating, the community becomes an integral part of the process, bringing their unique perspectives, knowledge, and resources to the table. This inclusive approach fosters a sense of ownership and collaboration, ultimately enhancing the effectiveness and sustainability of the intervention |  |  |  |
| **West Gojam, Amhara** |  |  |  |
| A significant number of individuals from the community may be encouraged to visit the health facility for TB screening and treatment. However, it is important to consider the potential consequences, such as an increased workload for healthcare workers who are already overwhelmed, as well as the strain on testing kits and drugs. Therefore, it is crucial to undertake sufficient preparation to address these challenges effectively-Hospital head | - Community Engagement, - Workload, - Need for resource consideration, - Preparation and addressing challenges, - Realities and challenges, - Addressing of challenges, - Barriers to healthcare access | ***Considering cross-cutting multiple factors*** |  |
| It is important to acknowledge that certain realities may remain unchanged despite the efforts of health programs. For instance, the presence of congregated homes and communities, as well as health facilities that do not meet the required standards for providing quality health services for TB elimination, may pose challenges. While these factors may be beyond the immediate control of health programs, it is crucial to recognize and address them in order to effectively work towards the goal of TB elimination-ZHB representative. |  |  |  |
| **Wolyta Zone Health Buraeu CDC** |  |  |  |
| I have a cncern that our people are suffering from malnutrition, low awareness of the diseases itself and could not afford to come to the health facilities for TB treatment.” One of the heads of zonal disease prevention and control |  |  |  |
| **West Gojam, Amhara** |  |  |  |
| Once patients are aware of the importance of taking the medication daily and the complication of TB drug interrupting, they will not do so. Hence, it is usually preferable to assign family members or a friend as treatment supporter or convince him to take the medications by himself.” One of the heads of zonal disease prevention and control | - Importance of medication adherence, - Complications of TB drug interruption, - Treatment support, - Individual responsibility, - Role of treatment supporters | ***The importance of assigning TB treatment supporter*** |  |
